# Does an instructional video as a stand-alone tool promote the acquisition of practical clinical skills? A randomised simulation research trial of skills acquisition and short-term retention

**DOI:** 10.1186/s12909-024-05714-6

**Published:** 2024-07-02

**Authors:** Thomas Ott, Tim Demare, Julia Möhrke, Saskia Silber, Johannes Schwab, Lukas Reuter, Ruben Westhphal, Irene Schmidtmann, Sven-Oliver Dietz, Nina Pirlich, Alexander Ziebart, Kristin Engelhard

**Affiliations:** 1 Department of Anaesthesiology, University Medical Centerof the, Johannes Gutenberg-University Mainz, Langenbeckstr. 1, Mainz, 55131 Germany; 2 Institute of Medical Biostatistics, Epidemiology, and Informatics, University Medical Centerof the, Johannes Gutenberg-University Mainz, Obere Zahlbacher Str. 69, Mainz, 55131 Germany; 3 Department of Orthopaedics and Traumatology, University Medical Centerof the, Johannes Gutenberg-University Mainz, Langenbeckstr. 1, Mainz, 55131 Germany

**Keywords:** Instructional video, Clinical skill, Undergraduate students, Self-study, Intraosseous access

## Abstract

**Background:**

The effectiveness of instructional videos as a stand-alone tool for the acquisition of practical skills is yet unknown because instructional videos are usually didactically embedded. Therefore, we evaluated the acquisition of the skill of a humeral intraosseous access via video in comparison to that of a self-study with an additional retention test.

**Methods:**

After ethical approval, we conducted two consecutive studies. Both were designed as randomised controlled two-armed trials with last-year medical students as independent samples at our institutional simulation centre of a tertiary university hospital centre. In Study 1, we randomly assigned 78 participants to two groups: Vid-Self participants watched an instructional video as an intervention, followed by a test, and after seven days did a self-study as a control, followed by a test. Self-Vid ran through the trial in reverse order.

In Study 2, we investigated the influence of the sequence of the two teaching methods on learning success in a new sample of 60 participants: Vid-Self watched an instructional video and directly afterward did the self-study followed by a test, whereas Self-Vid ran through that trial in reverse order.

In Studies 1 and 2, the primary outcome was the score (worst score = 0, best score = 20) of the test after intervention and control. The secondary outcome in Study 1 was the change in score after seven days.

**Results:**

Study 1: The Vid-Self (Participants *n* = 42) was superior to the Self-Vid (*n* = 36) (mean score 14.8 vs. 7.7, *p* < 0.001). After seven days, Self-vid outperformed Vid-Self (mean score 15.9 vs. 12.5, *p* < 0.001).

Study 2: The Vid-Self (*n* = 30) and Self-Vid (*n* = 30) scores did not significantly differ (mean 16.5 vs. mean 16.5, *p* = 0.97).

**Conclusion:**

An instructional video as a stand-alone tool effectively promotes the acquisition of practical skills. The best results are yielded by a combination of an instructional video and self-study right after each other, irrespective of sequence.

**Trial registrations:**

ClinicalTrials.gov: NCT05066204 (13/04/2021) (Study 1) and NCT04842357 (04/10/2021) (Study 2).

**Supplementary Information:**

The online version contains supplementary material available at 10.1186/s12909-024-05714-6.

## Background

### Instructional videos

Instructional videos are increasingly applied in medical education.[1–4] The advantage of instructional videos, in contrast to lectures and face-to-face teaching, is greater flexibility in learning when provided independently of time.[1, 2, 5] The need for distance learning during the COVID-19 pandemic as well as the aspired individualisation and flexibility of learning within curricula foster the intensified expansion of online teaching and particularly instructional videos.[6–8] Instructional videos have a positive effect on knowledge.[9] However, their impact on the acquisition of practical skills is controversial due to inconsistent results.[10–14] Moreover, previous studies evaluated instructional videos in comparison to face-to-face teaching, or the videos were didactically embedded, which means that they were also implemented to tutor practical training and were applied repetitively.[10–12] To our knowledge, no study has focused on instructional videos as a stand-alone tool without didactic embedding. If instructional videos prove to be effective as stand-alone tools, crucial implications could be deduced for their deliberate application in medical school concerning distance learning, standardisation and flexibility.

### Intraosseous access

To evaluate the value of an instructional video as a stand-alone tool on technical skills acquisition, two factors must be considered. First, it is advantageous to use a procedure that is essential for patient care and that can be devided into well-defined steps. Second, it should be a procedure that has received little attention in curricula to reduce bias concerning previous experience of the participants. [15–17] Therefore, we chose to apply intraosseous access (IOA) to the humeral head. IOAs show high success rates in patients and can be effectively trained using skill trainers.[18–22] For emergencies, the application of an IOA is most common at the proximal tibial plateau and less common at the humeral head.[20, 23] In the case of contraindications to accessing the tibia, a humeral IOA must be mastered as an alternative. Interestingly, humeral IOA training has received less attention in the literature than tibial IOA training.[19, 24] Therefore, we produced a ten-minute instructional video on humeral IOA for adult emergency patients and evaluated its effect on students.[25].

### Hypotheses

In Study 1, we evaluated the effect of this video by comparing the intervention ‘*INSTRUCTIONAL video’* to the control *‘self-study’* on the acquisition of the skill in two study groups. The performance was quantified by a test that results in a score. Our null hypothesis with respect to the primary endpoint was as follows: The group that watched an instructional video did not differ in score from the group that did self-study at the same time. To evaluate skill retention as a secondary endpoint and to ensure the same overall training experience for both groups, we repeated that trial seven days later in reverse order of both groups. The results of the secondary endpoints of *Study 1* yielded findings that are described below and are worthy of further evaluation. Therefore, six months later, we recruited a new sample with similar demographic characteristics and defined this new investigation as *Study 2*. In this study, the instructional video and self-study were conducted directly after each other, and participants were tested directly afterwards. Only the order of the teaching methods differed between the two groups. The null hypothesis was formulated as follows: The group that watched an instructional video before self-study did not differ in score from the group that watched an instructional video after self-study.

## Methods

### Ethical aspects

The responsible ethics committee (Ethical Review Committee of the State Chamber of Physicians of Rhineland-Palatinate, Deutschhausplatz 3, 55,116 Mainz, Germany; Chairperson: Professor S. Letzel) approved Study 1 on 29. April 2021 under 2021–15807 and Study 2 on 21. October 2021 under the number 2021–16112. Participation was voluntary, and written informed consent was signed before participation.

### Study design

**Fig. 1:**
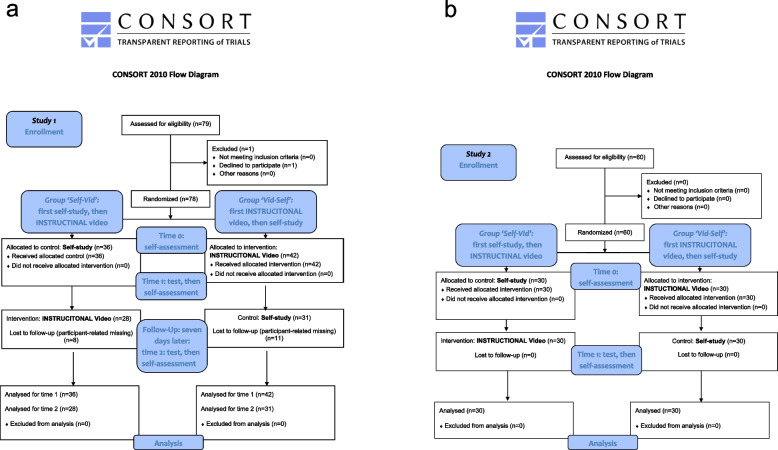
**a** CONSORT flow chart of Study 1. In Study 1, the Self-Vid group first had to complete a self-assessment (T0), then had to perform the self-study and was tested afterwards, followed by a second self-assessment (T1). After seven days, the group watched the instructional video, was tested, and had to repeat the self-assessment for the third time (T2). The Vid-Self group first completed a self-assessment (T0) and then watched the instructional video, followed by the test and the second self-assessment (T1). After seven days, the group performed the self-study, was tested, and then, the self-assessment was repeated for the last time (T2). **b** CONSORT flow chart of Study 2. In Study 2, both groups had to complete a self-assessment, and then the instructional video and self-study were conducted in immediate succession according to group assignment. After that, a self-assessment was performed again

We conducted two prospective randomised controlled two-armed simulation-based research studies as investigator-initiated trials with independent samples, aiming for a 1:1 ratio concerning the number of participants in each group. Study 1 included three points in time, and Study 2 included two points in time (Fig. 1a, b).

### Participants, previous experience and setting

We recruited 78 last-year medical students for Study 1 and an additional 60 students for Study 2. The studies were conducted during the mandatory institutional final year training at our institutional simulation centre. One year before this training, all participants attended a curricular 20-min session of face-to-face practical training without video presentation or standardised didactic conception to a maximum of five students at a time concerning IOA located in the tibia; students applied the device several times, but without a defined number of attempts. The same device and skill trainer were used in the present study.

Study 1 was conducted in May and June, and Study 2 was conducted in November and December 2021.

### Intraosseous equipment

We used the Arrow EZ-IO Intraosseous Vascular Access System (Teleflex Medical Europe Ltd., Athlone, Ireland) with cannulas of three sizes. As a skill trainer, we used the EZ-IO humeral training bone (Teleflex Medical Europe Ltd., Athlone, Ireland) for a maximum of 5 attempts. As all participants had undergone curricular IOA-training one year before the study no further familiarisation was deemed necessary and hence none was provided.

### Measuring tools

#### Test

The test was videotaped for evaluation. Participants were put in front of a video camera (Lumifix F2000, Panasonic, Kadoma, Japan) that pointed from the participant´s shoulder to a table containing the IOA equipment. First, participants demonstrated and explained the location of humeral IOA on their own extremity; they wore scrubs to do this. Then, the participants prepared the equipment and performed the IOA in the skill trainer. The performance of the students was assessed by a score that was designed and tested by our study group.

#### Score

Currently, there are no validated checklists for assessing humeral IOA. Hence, authors who are experienced in IOA in patients performed five rounds of focus group sessions according to Schutz et al. and adapted an already validated score for tibial IOA to the needs of the present study.[26, 27] The resulting checklist consisted of 15 weighted items quantifying the performance of humeral IOA and is cited in additional file 1. The sum of the particular items results in a score of 0 (worst performance) or 20 (best performance). The entire procedure is described in detail in additional file 2.

#### Evaluation of the test

Two authors (TD, JM) evaluated the videotapes as raters in a randomised sequence and were blinded to the participants’ group assignment and the time points that are described in the following section. The videotapes were observed by both rates simultaneously using Windows Media Player (Windows X, Microsoft, Redmond, USA). After watching each individual video they discussed discrepancies thoroughly and agreed on one score per videotape.

#### Self-assessment

Participants had to self-estimate their general capability of applying an IOA on a scale from 1 (very good) to 6 (very bad) as a global rating scale.[28].

### Intervention and control

#### Intervention: instructional video

A ten-minute instructional video about humeral intraosseous access was produced by the authors according to the current literature and the manufacturer’s instructions. An identical device and skill trainer were used for the instructional video and the test. Participants individually watched this video on an iPad (iPad Pro 2. Gen., Apple, Silicon Valley, USA) in a quiet room during the mandatory training.

#### Control: self-study

The self-study included ten minutes of unsupervised hands-on exercise with the device and the skill trainer in separate rooms. No further instructional materials were provided.

### Data collection

Randomisation was performed and controlled by certain authors (TD, JM, SS, JS, and LR). Participants were randomly allocated into one of two groups by drawing a lot from an opaque box in Study 1. In Study 2, separate opaque boxes for male and female participants were provided, thus allowing us to stratify the randomisation by sex due to gender differences that were observed in Study 1 and are detailed in the Results section. Participants were instructed not to disclose information on their allocation before everybody had drawn their lots, thus ensuring allocation concealment. The two study groups were:

#### The ‘Vid-Self’ group (first, INSTRUCTIONAL video; second, self-study)

In Study 1, participants in the ‘Vid-Self’ group first watched the instructional video, subsequently took the test and performed the self-assessment. Seven days later, they performed a 10-min self-study and subsequently took the test and the self-assessment again.

In Study 2, participants in the ‘Vid-Self’ group watched the instructional video and then did the self-study immediately afterwards. Then they did the test and then performed the self-assessment.

#### The Self-Vid group (first self-study, second INSTRUCTIONAL video)

In Study 1, participants in the ‘Self-Vid’ group first performed a 10-min self-study and then took the test. Seven days later, they watched the instructional video and subsequently took the test and self-assessment.

In Study 2, participants in the ‘Self-Vid’ group performed self-study first, watched the instructional video, did the test and then performed the self-assessment.

The data were collected at three consecutive points in time (T) in Study 1. At T0, randomisation was performed, and the participants’ demographic information, previous experiences and self-assessment were collected. At T1, participants underwent the *intervention* or *control* and then took the test and self-assessment. At T2 (retention), seven days after T1, the groups were switched between *intervention* and *control*, after which the test and self-assessment were performed.

The data were collected at two consecutive points in time (T) in Study 2: T0 was identical to that in Study 1. At T1, participants had already performed the self-study and watched the instructional video in a randomised order, and then took the test and self-assessment.

### Sample size considerations

For Study 1, initially we had planned pre-post-comparisons to evaluate the individual learning success in each sequence group. For this, based on the publication of Oriot et al., [26] we had assumed an improvement from the level of inexperienced participants (mean 11.06; standard deviation (SD) 4.08) halfway to the level of experienced physicians (mean 19,13; SD 1,48) and a correlation of 0.5 between both measurements. For a two-sided paired t-test to establish this improvement at the 5% significance level with 80% power, 11 participants in each group were required. However, we changed our study design due to concerns that setting a preliminary test before any study might influence students learning efforts too much. Therefore, we decided to omit the preliminary test and to focus on the comparison between instructional video and self-study as a first learning exposure as our primary endpoint. This lead to considering a difference of 3 points in the score as relevant and assumed a standard deviation of 4 based on the publication of Oriot et al., [26] which resulted in an effect size of 0.75. To obtain a power of 90% to detect such an effect at the 5% level with a two-sided two-sample t-test two groups of 39 students each were required.

For Study 2, we used our data from Study 1. The observed means and standard deviations resulted in an effect size of 1.14. Using a two-sided two-sample t-test, such an effect could be established at the 5% level with 80% power if 14 students per group were included. However, more students were interested in taking part and we did not want to exclude anybody. Therefore, actually 60 students were included in study 2. Thus, the actual sample size was sufficient to reproduce the effect of study 1 if the effect of the sequence of learning methods within a short period is indeed the same as the effect of the sequence of learning methods with a gap of one week and first test after the first learning sequence.

### Statistics

For both studies, we performed intention to treat analyses and included all participants with available test results. For quantitative data, the score of each group at each point in time was quoted as the mean and SD and displayed as a boxplot. For Study 1, the differences within groups are also reported as the mean and SD.

To test for differences between the Vid-Self group and Self-Vid group, a two-sided two sample t-test was performed for the primary endpoint: the difference in the sum of scores at T1 between the groups in both studies. All the other tests applied to the analysis of the secondary endpoints were exploratory; therefore, no correction for multiple testing was applied. In Study 1, we performed a two-sided two sample t-test for differences in scores *between the two groups at T2.* We performed paired t-tests for differences in scores *within each group (dependent samples) between* T1 and T2. To make test scores and self-assessments, which were measured on different scales, comparable, we standardised the variables in both studies by subtracting the mean for the complete sample from each score and dividing it by the standard deviation (SD) and computed the difference between the two standardised measurements. Small differences correspond to consistency of self-assessment and score, large differences correspond to inconsistency. We then tested for differences of these differences between genders with a two-sided two sample t-test.

## Results

In Study 1, 78 participants were tested at T1: 42 (54%) participants were assigned to Vid-Self, and 36 (46%) were assigned to Self-Vid. At T2, 59 participants were analysed, as 21 participants did not appear: 31 (53%) participants were evaluated in the Vid-Self group, and 28 (48%) in the Self-Vid group. In Study 2, 30 of 60 (50%) participants were assigned to each study group, and all were analysed. The demographic data are shown in Table 1. (Table 1 see below).

**Table 1:** Demography of the participants separated by study group: age, sex and previous experience. The data of the participants in both groups are expressed as the number (n) and ratio (%) of all participants in one group. In Study 1, the Vid-Self group comprises *n* = 42, and the Self-Vid group comprises *n* = 36. In Study 2, the Vid-Self and Self-Vid group constitute *n* = 30 each. The sum of the percentages of one item occasionally exceeded 100 due to mathematical rounding

***Study 1***		**Vid-Self**	**Self-Vid**
		Number of participants (percentage of the group^a^)	Number of participants (percentage of the group^a^)
Age in years	Median SD	28.2 (100) 3.8	28.6 (100) 3.3
Sex	female	25 (60)	19 (53)
	male	17 (40)	17 (47)
Experience additional to medical curriculum	none	20 (48)	20 (56)
	EMT^b^	2 (5)	0
	paramedic	11 (26)	10 (28)
	nurse	9 (21)	5 (14)
	intensive care nurse	0 (0)	1 (3)
Previously trained in intraosseous access	Yes	16 (38)	13 (36)
	No	26 (62)	23 (64)
Intraosseous accesses performed in patients	None	36 (86)	34 (94)
	1	1 (2)	1 (3)
	2–5	5 (12)	1 (3)
Humeral intraosseous accesses performed	No	40 (95)	36 (100)
	Yes	2 (5)	0 (0)
Numbers of Intraosseous accesses observed	0	30 (71)	23 (64)
	1	3 (7)	8 (22)
	2–5	3 (7)	4 (11)
	6–10	3 (7)	0
	> 10	2 (5)	2 (6)
Dropout at T2		11 (26)	8 (22)
***Study 2***			
Age in years	Median	27.9 (100)	27.6 (100)
	SD	2.8	3.3
Sex	female	15 (50)	15 (50)
	male	15 (50)	15 (50)
Experience additional to medical curriculum	none	8 (27)	10 (33)
	EMT^b^	3 (10)	2 (7)
	paramedic	6 (20)	9 (30)
	nurse	4 (13)	4 (13)
	intensive care nurse	9 (30)	5 (17)
Previously trained in intraosseous access	Yes	18 (60)	21 (70)
	No	12 (40)	9 (30)
Intraosseous accesses performed in patients	None	23 (78)	21 (70)
	1	0	0
	2–5	5 (17)	8 (27)
	6–10	1 (3)	0 (0)
	> 10	1 (3)	1 (3)
Humeral intraosseous accesses performed	No	30 (100)	30 (100)
	Yes	0 (0)	0 (0)
Numbers of Intraosseous accesses observed	0	20 (67)	16 (53)
	1	1 (3)	0 (0)
	2–5	6 (20)	9 (30)
	6–10	1 (3)	3 (10)
	> 10	2 (7)	2 (7)

^a^The sum of the percentages is unequal to 100 due to mathematical rounding

^b^Emergency medical technician

### Primary endpoint of study 1

**Fig. 2:**
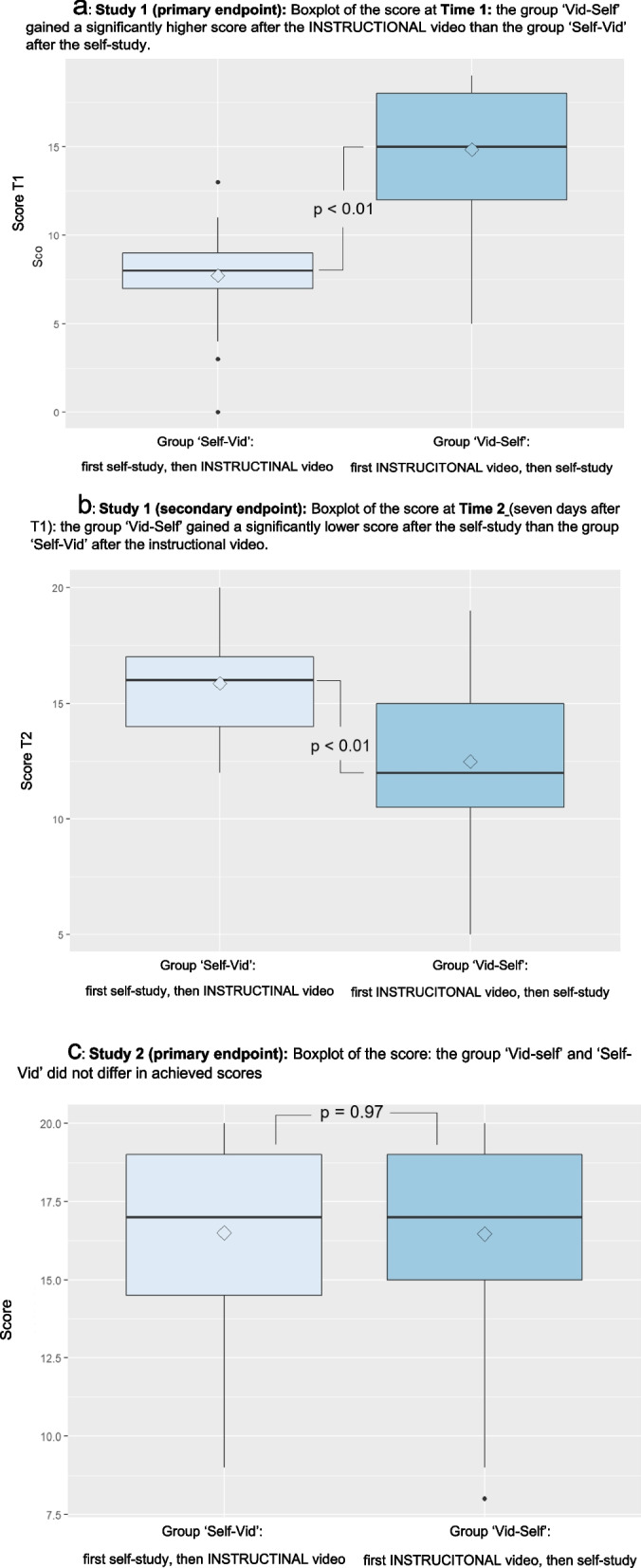
Boxplot of the scores of Study 1 and Study 2. These boxplots display the scores of the two groups on the abscissa: The Self-Vid group and the Vid-Self group. The ordinate shows the score with a minimum of 0 and a maximum of 20. The horizontal thick lines indicate the median, the thin lines indicate the first and third quartiles, and the vertical bars indicate the minimum and maximum scores. The dots indicate extreme values, and the rhombs indicate the means of the scores. **a** Boxplot of the scores in Study 1 at T1. Study 1 (primary endpoint): Boxplot of the score at Time 1: The ‘Vid-Self’ group obtained a significantly greater score after the INSTRUCTIONAL video than did the ‘Self-Vid’ group after the self-study. (primary endpoint of Study 1: *p* < 0.01). **b** Boxplot of the scores in Study 1 at T2. Study 1 (secondary endpoint): Boxplot of the score at Time 2 (seven days after T1): The ‘Vid-Self’ group had a significantly lower score after the self-study than did the ‘Self-Vid’ group after the instructional video (secondary endpoint of Study 1: *p* < 0.01). **c** Boxplot of the scores in Study 2. Study 2 (primary endpoint): Boxplot of the score: The ‘Vid-self’ and ‘Self-Vid’ groups did not differ in terms of the achieved scores (*p* = 0.97)

In Study 1, the group that watched the instructional video at that point in time scored significantly greater than the group that did self-study (Self-Vid at T1) (at T1: Vid-Self: mean 14.8, SD 3.5 vs. Self-Vid: mean 7.7, SD 2.6, *p* < 0.001) (Fig. 2a, additional file 3).

### Secondary endpoints of study 1

In Study 1, at T2 (after seven days), Vid-Self tended to yield lower scores than Self-Vid (mean 12.5, SD 3.6 vs. mean 15.9, SD 2.2, *p* < 0.001) (Fig. 2b). From T1 to T2, the scores tended to decrease for Vid-Self (T1: mean 14.8, SD 3.5; T2: mean 12.5, SD 3.6, *p* < 0.001) and increase for Self-Vid (T1: mean 7.7, SD 2.6, *p* < 0.001; T2: mean: 15.9, SD 2.2, *p* < 0.001).

The absolute value of the score of Vid-Self tended to decrease less than the score increased in Self-Vid (mean change from T1 to T2:—2.8 vs. 7.9, *p* < 0.001).

**Fig. 3:**
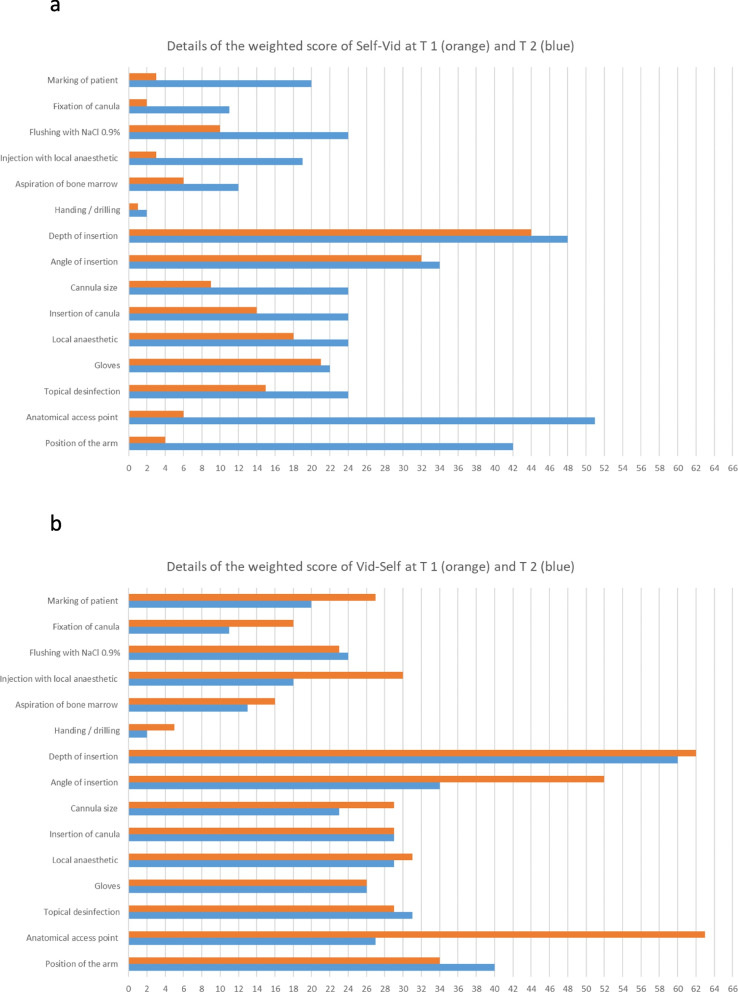
Details of the weighted scores of participants in Study 1. **a **The diagram contains the data of participants of the Vid-Self group in Study 1 who performed the tests at both points in time (*n* = 31). For those participants, the diagram displays the sum of the single items of the score with respect to their weighting, as described in the “Methods” section. The values of the single items were weighted from 1 to 3 concerning the maximum number of achievable points according to their impact on clinical relevance (additional file 1). For example, the maximum score for the item “Anatomical access point” was 3 points, so for 31 participants it was were equivalent to 93 points. The points in time are displayed as follows. T 1: orange, T 2: blue. **b **The diagram contains data from participants of the Self-Vid group in Study 1 who performed the test at both points in time (*n* = 24)

The details of the individual weighted items of the scores of those participants attending T1 and T2 are shown in Fig. 3.

We investigated whether these changes in score within the seven days could be explained by the sequence (video then self-study or vice versa) or represented a decrease in skill in study 2.

In study 1 Vid-Self compassed 19 female and 17 male participants whereas Self-Vid compassed 25 female and 17 male participants.

Separated by gender, female and male participants did not exhibit substantial differences in score over both groups (T1: mean: 11.2, SD 4.8 vs. mean 12.0, SD 4.6, *p* = 0.459; T2: mean 13.3, SD 3.8 vs. mean 14.9, SD 2.9, *p* = 0.069, additional file 3).

Separated by gender and study groups at T1 female and male participants did not show a difference either (additional file 3). At T2 female participants of Vid-self tended to show a lower score than male (female mean: 10.9, SD 3.1, male mean: 14.4, SD 3.3, p = 0.007) whereas there was no difference at T2 between sexes in Self-Vid (additional file 3).

Concerning self-assessment female participants generally tended towards a worse self-assessment than male (T0: *p* < 0.001, T1: *p* = 0.027, T2, *p* = 0.001, additional file 3).

Separated by gender and study groups at T0 female participants of the Vid-Self group tended to show a worse self-assessment than male participants whereas in the Self-Vid group sexes did not exhibit differences in self-assessment (Vid-Self: *p* = 0.002, Self-Vid: *p* = 0.1, additional file 3).

When addressing gender differences in consistency of self-assessment and score, a significant difference of differences between females and males was observed only at T2 in the Self-Vid group (*p* = 0.049); moreover, there was no difference in the other points over time (particular *p* > 0.05). Due to this sex difference at T2, we stratified for sex in Study 2.

### Primary endpoint of study 2

In Study 2, neither group differed in score (Vid-Self: mean 16.5, SD 3.0 vs. Self-Vid: mean 16.5, SD 3.1, *p* = 0.97) (Fig. 2c).

### Secondary endpoints of study 2

In Study 2, self-assessments were recorded for the Vid-Self group (mean 4.5, SD 1.2; mean 2.9, SD 0.9), and the Self-Vid group (mean 4.1, SD 1.1; mean 2.5, SD 0.9). Again, female and male participants did not exhibit substantial differences in score (mean 16.8, SD 2.8 vs. mean 16.2, SD 3.2, *p* = 0.417). Again, male participants tended to have slightly better self-assessments than did their scores, while the opposite trend was observed for female participants, but the difference was not statistically significant (*p* > 0.1). An overview of the entire results is provided in additional file 3.

## Discussion

Two studies showed that an instructional video, as a stand-alone tool without didactic embedding, promoted the acquisition of practical clinical skills. Furthermore, participants generally obtained the highest scores after watching the instructional video (Vid-Self group: 14.8 points on day one; Self-Vid group: 15.9 points on day seven). In comparison, the participants performed significantly worse directly after self-study (Self-Vid group: 7.7 points on day one; Vid-Self group: 12.5 points on day seven). The decline in score in Study 1 over seven days in the Vid-Self group suggested that there was a short-term decline in this skill, even though self-study was performed directly before the test. The follow-up study (Study 2) showed that, regardless of the sequence of the skill acquisition methods (self-study or video), the immediate combination of the two skill acquisition methods was most successful, as both groups scored 16.5 points (Fig. 2b). We deduce that an instructional video as a stand-alone tool effectively promotes the acquisition of this practical skill, and self-study even fosters that acquisition.

### Acquisition of practical skills

Traditionally, practical skills were taught face-to-face in group sessions. Due to the pandemic, groups had to be reduced in size, which required an increased number of instructors as well as sessions. Therefore, recently, alternative teaching methods such as instructional videos have been more frequently integrated into medical education.[1–3, 6, 7] Instructional videos teach identical content in a cross-sectional and longitudinal manner and therefore may ensure more standardisation of a specific content than face-to-face instruction.[1, 3] A previous study evaluated the effect of a ten-minute video followed by ten minutes of untutored training in comparison to 20 min of face-to-face instruction concerning paediatric tibial IOA.[12] The video group scored significantly higher on the subsequent test than did the control group (7.56 vs. 6.00, maximum possible: 10). Although the latter study included a smaller but more highly qualified sample, the present study showed similar results for inexperienced participants. Another previous study evaluated three teaching methods concerning subcuticular suturing but in an elaborate didactic embedding procedure involving eight minutes of video, face-to-face instruction and independent practice.[10] The main difference from our study was that those participants watched the video first and subsequently were randomised into the cited groups. Furthermore, the video group repeatedly watched the video. However, as in our study, video promoted the acquisition of the skill, as did instructor-led training, whereas independent practice was less effective. However, the present study revealed that an instructional video as a stand-alone tool can teach practical skills well without additional didactic embedding or extensive previous experience. To optimise learning success, a combination of an instructional video with self-study is recommended, independent of the sequence of both teaching methods.

### Skill decline

A decrease in clinical skills depends on affective, cognitive, and psychomotor aspects, time, frequency of practice, and prior experience. [29–32] Over a 12-month period, experienced providers show a decline in the skill of accessing IOA, as do undergraduates in basic life support.[30, 31] Furthermore, experienced providers show better retention of internal pacemaker placement skills over a three-month period than inexperienced physicians.[32] In novices, the ability to perform paracentesis decreases within three months, and the ability to perform endoscopic intubation decreases within two months. The performance of focused transthoracic echocardiography and suturing decreases within one month.[11, 33–35] Only the above cited study described a decrease in skill concerning subcuticular suturing within one week.[10] The group that was trained by a video declined less (12.74 to 12.41) than the instructor trained (14.17 to 13.00) and the independent practice group (13.54 to 11.2) [10]. As mentioned above, the videos in that study were used repetitively. Therefore, participants were exposed to more video experience than in the present study. Future trials should focus on how repetitive videos foster skill retention.

To explore this decrease in skill, we analysed the development of single items in our score (additional file 1) in both groups (Fig. 3). The score consists of 15 weighted items and a maximum score of 20 (additional file 2). Figure 3 shows the sum of the scores for each item and its weights. In the Vid-Self group in Study 1, the decrease in score from T1 to T2 was based mainly on the following items: anatomical access (weighted: 3), angle of insertion (weighted: 2), injection of local anaesthetic, fixation of the cannula, and marking of the patient (weighted: 1 each). The first two items are clinically relevant for successfully applying an IOA. These factors appear to contribute most to the decline. In the Self-Vid group, the increase in scores was caused mainly by the same items and also by the item arm position. Therefore, in our opinion, the score adequately reflects performance in terms of relevant clinical aspects. Furthermore, the cited items of the score seem to be efficiently taught via an instructional video.

### Gender aspects

In Study 1, we noticed trends, however, without a statistically significant difference: Female participants tended to have a lower mean score in all groups. Due to the greater proportion of female participants in the Vid-Self group who had a lower score after seven days, this could be a confounder or a gender issue. The latter has been controversially discussed in many fields of medicine.[36–39] Furthermore, males in the Self-Vid group had better self-assessments than did their performance, while females had worse self-assessments than did their performance. Therefore, we stratified patients by sex in Study 2. However, there was no statistically significant difference concerning sex in Study 2.

### Limitations

First, any simulation-based study has limitations due to the artificial environment. Therefore, the results should be interpreted with caution concerning possible transferability in patient care, and generalizability is limited to laboratory conditions.[11, 12] Second, we found no validated score for the evaluation of humeral IOA; therefore, we thoroughly performed the adoption of this validated score for tibial access (additional file 2) according to an established procedure.[27] We partially used weighted items within this score that may influence the achieved score disproportionally high concerning the particular item and we did not perform a statistical validation. However, we developed our score out of a validated score and estimated this as appropriate for our needs. Further validation is worthwhile. Third, although all students attended a curricular training in intraosseous vascular access one year before the study 49 of 78 (62%) participants in study 1 and 21 of 60 (35%) in study 2 stated not to have had any training before. Apparently, this training had no substantial impact on the participants and further studies should include familiarisation with the equipment used. Fourth, a dropout in Study 1 of 27% (21/78) of the participants in the follow-up at Time 2 (seven days after Time 1) was quite high. This was probably caused by the organisational effort of those participants being engaged in remote hospitals to attend the follow-up. However, dropout may have caused an imbalance in the sex ratio at time 2, influencing us to reevaluate our findings in Study 2, as discussed above. Fifth, self-study as a control instrument seems to be trivial because teaching is certainly better than not teaching. Nevertheless, our aim was to evaluate a video as a stand-alone tool, so we needed the best possible inert control group. All participants had already completed curricular IOA training for the tibial access site one year before the study. Therefore, we decided not to perform a pretest concerning the video, as in previous studies, but rather defined self-study as the best possible control for contrasting the effect of the video.[10, 11].

## Conclusions

A practical skill can be efficiently acquired by an instructional video as a stand-alone tool without didactic embedding and is superior to self-study despite previous curricular experience. Therefore, instructional videos can be used to a satisfactory extent for skill acquisition when direct teaching is impossible, such as during a pandemic. A decline in performance can be observed within seven days after the instructional video, which cannot be prevented even by self-study immediately before testing. However, the best results could be achieved by the immediate combination of instructional video and self-study. Hereby, the sequence of the methods has no influence on the acquisition. Gender differences could not be detected in the present studies. The evaluated instructional video proved to be a stand-alone tool for the acquisition of the defined practical skill. Instructional videos could greatly increase the efficiency of teaching in medical schools and provide a useful supplement to students' education.

### Supplementary Information

Supplementary Material 1.

Supplementary Material 2.

Supplementary Material 3.

## Data Availability

The dataset supporting the conclusions of this article is available in the LabArchives repository, 10.25833/8cc7-eb07 at 10.25833/8cc7-eb07 The raw data were anonymised according to the protocols of the present study.
