# Supplementary material for: Does an instructional video as a stand-alone tool promote the acquisition of practical clinical skills? A randomised simulation research trial of skills acquisition and short-term retention
Source: BMC Med Educ. 2024 Jul 2;24:714. doi: 10.1186/s12909-024-05714-6 (PMC11221112; doi:10.1186/s12909-024-05714-6)
Supplement: Supplementary file 1 — Supplementary Material 1. [file 12909_2024_5714_MOESM1_ESM.docx]

| **1. Position of the arm** | Not correct | 0 |
| --- | --- | --- |
|  | correct | 2 |
| **2. Anatomical** | Not correct | 0 |
| **access point** | Correct | 3 |
| **3. Topical desinfection** | Not used | 0 |
|  | Used | 1 |
| **4. Gloves** | Absence of gloves | 0 |
|  | Sterile gloves | 1 |
| **5. Local anaesthetic** | Not used | 0 |
|  | Used (awake patient) | 1 |
| **6. Insertion of cannula** | Insertion while drilling | 0 |
|  | Insertion until bone contact without drilling | 1 |
| **7. Choice of cannula size** | Canuala exceeds skinsurface more than  1 cm | 0 |
|  | Canula exceeds surface of skin less than  1 cm | 1 |
| **8. Angle of insertion** | Oblique ankle | 0 |
| **(prove after completion)** | 90 degrees to surface of the skin | 2 |
| **9. Depth of insertion** | Canula exceeds skin surface more than  1 cm | 0 |
|  | Canula exceeds surface of skin less than  1 cm | 2 |
| **10. Handing / drilling** | Not correct drilled | 0 |
|  | 1 cm advancement of the canula drilling after visible loss of resistance | 1 |
| **11. Aspiration of bone** | Not performed | 0 |
| **marrow** | Performed | 1 |
| **12. Injection of local** | Not performed | 0 |
| **anaesthetic** | Performed | 1 |
| **13. Flushing with** | Not performed | 0 |
| **NaCl 0.9%** | Performed | 1 |
| **14. Fixation of canula** | Not considered | 0 |
|  | Considered | 1 |
| **15. Marking of patient with** | Not considered | 0 |
| **a batch** | Considered | 1 |
| **Sum of points** |  |  |

| Raters | |
| --- | --- |
|  |  |
