# Supplementary material for: Does an instructional video as a stand-alone tool promote the acquisition of practical clinical skills? A randomised simulation research trial of skills acquisition and short-term retention
Source: BMC Med Educ. 2024 Jul 2;24:714. doi: 10.1186/s12909-024-05714-6 (PMC11221112; doi:10.1186/s12909-024-05714-6)
Supplement: Supplementary file 2 — Supplementary Material 2. [file 12909_2024_5714_MOESM2_ESM.docx]

***Additional file 2 – Development of the score***

Development of the score applied for evaluation of the performance in five rounds of focus groups according to Schmutz et al. (31)

**Focus group round 1**

The publication of Oriot et al. and the single items of the score (item 1 to 12) were analysed (Table A1).(30) Those items applicable for our study were identified: 2, 3, 4, 7, 8, 12 and those needing adaption: 1, 5, 6, 9, 10, 11, concerning localisation of access site, applied device and adjuncts, use of local anaesthetic and patients marking.

**Table A1: Original score for assessment of IO placement in the tibia according to Orit et al. 2012 (30)**

| **Nr** | **Item** | **Selection** | **Points weighted)** |
| --- | --- | --- | --- |
| 1 | Position of the nee | Unflexed | 0 |
|  |  | Flexed | 1 |
| 2 | Topical antiseptic | Not used | 0 |
|  |  | Used on the puncture site | 1 |
| 3 | Gloves | Absence of gloves or nonsterile gloves | 0 |
|  |  | Sterile gloves | 1 |
| 4 | Local anesthesia | Absence of anesthesia (in conscious patient) | 0 |
|  |  | Local anesthesia | 1 |
| 5 | Safety guard on needle | Absence of guard or guard >1.5 cm | 0 |
|  |  | Safety guard of 1 cm with thumb-index | 2 |
| 6 | Insertion technique | Absence of twisting motion | 0 |
|  |  | Mild twisting motion (1 or 2 moves) or no axial insertion | 1 |
|  |  | Axial twisting motion | 3 |
| 7 | Fluid aspiration from marrow cavity with an empty syringe | Absence of aspiration | 0 |
|  |  | Aspiration of fluid | 2 |
| 8 | Infusion of 0.9% NaCl | Absence of Infusion of 0.9% NaCl | 0 |
|  |  | Infusion of 0.9% NaCl | 2 |
| 9 | Securing the line | Absence of taping | 0 |
|  |  | Taping with line twice or more on limb and/or abdomen | 1 |
| 10 | Stability of the leg | Absence splint | 0 |
|  |  | Splint from knee to ankle | 1 |
| 11 | Location of the puncture site (observed after insertion) | Outside the puncture site or mobile needle | 0 |
|  |  | On the puncture site +/- 0.5 cm | 3 |
| 12 | Angle of insertion (observed after insertion) | Oblique insertion | 0 |
|  |  | Perpendicular insertion +/- 10 degree | 2 |
| Maximum sum: 20 | | | |

Then, the particular item of the score of Oriot et al 2012 were modified according to the current study demands (Table A2).

**Table A2: Modification of the particular score items**

| **Nr** | **Item** | **Adapted to** | **Reason of modification** |
| --- | --- | --- | --- |
| 1 | Position of the nee | Position of the arm: forearm placed vertically over the chest, or straight along the lateral margin of the patient in a maximal pronated position | Different localisation of access |
| 5 | Safety guard on needle | Depth of insertion | 3 sizes of cannulas |
| 6 | Insertion technique | Handling and drilling | A battery driven equipment was used so that no manual twisting is applicable in the present study |
| 10 | Stability of the leg | Fixation of cannula | There is no splinting recommended in the humeral access |
| 11 | Location of the puncture site (observed after insertion) | Localisation of puncture site | localisation was modified from tibial to humeral |

**Focus group round 2**

The items were chronologically sequenced according to current recommendations and the study setting. The weighting of the single items according to original score were discussed and adapted (Table A3).

**Table A3: Chronological sequencing of the items according to the current study design**

| **Nr** | **Item** | **Selection** | **Weighting** |
| --- | --- | --- | --- |
| 1 | Position of the arm | Not correct | 0 |
|  |  | Correct | 1 |
| 2 | Anatomical access point | Not correct | 0 |
|  |  | correct | 3 |
| 3 | Topical desinfection | Not used | 0 |
|  |  | Used | 1 |
| 4 | Gloves | Absence of gloves | 0 |
|  |  | Sterile gloves | 1 |
| 5 | Local anaesthetic | Not used | 0 |
|  |  | Used (awake patient) | 1 |
| 6 | Insertion of cannula | Insertion while drilling | 0 |
|  |  | Insertion until bone contact without drilling | 1 |
| 7 | Choice of cannula size | Too short | 0 |
|  |  | 4.5 cm cannula | 2 |
| 8 | Angle of insertion (prove after completion) | Oblique angle | 0 |
|  |  | 90 degrees to surface of the skin | 2 |
| 9 | Depth of insertion after intraosseous insertion | Cannula penetrates the bone less  than 1 cm | 0 |
|  |  | Cannula penetrated bone more than  1 cm | 1 |
| 10 | Handing / drilling | Persistent drilling | 0 |
|  |  | Short drilling until cannula penetrates the bone after | 1 |
| 11 | Aspiration of bone marrow | Not performed | 0 |
|  |  | performed | 2 |
| 12 | Injection of local anaesthetic | Not performed | 0 |
|  |  | Performed | 1 |
| 13 | Flushing with NaCl 0.9% | Not performed | 0 |
|  |  | Performed | 2 |
| 14 | Fixation of cannula | Not considered | 0 |
|  |  | Considered | 1 |
| 15 | Marking of patient with a batch | Not considered | 0 |
|  |  | Considered | 1 |
| Maximum sum: 21 | | | |

**Focus group round 3**

The new score was pilot tested: The authors observed an inexperienced and an experienced volunteer physician during IOA performance. Following, further adaptions were necessary concerning drill device handling and insertion depth. The weighting was reviewed and a maximum sum of 20 resulted (table A4).

Considerations that were discussed:

Item: 1: Position of the arm: this is a very important item, weighted with 2 due the possible damage to the biceps tendon if the arm is not positioned correctly.

Item: 2: Anatomical access point: this is a key issue of bone marrow puncture, therefore weighted 3.

Item 8: as to the original publication, an oblique penetration of the one probably fails the bone marrow, therefore weighted 2.

Item 12 and 13: we applied a local anaesthetic to the bone marrow before flushing the intraosseous access. So, we have an additional test to verify the correct position of the cannula and weighted flushing and application of local anaesthetic 1 point each.

**Table A4: Adapted score defined for study purpose**

| **Nr** | **Item** | **Selection** | **Weighting** |
| --- | --- | --- | --- |
| 1 | Position of the arm | Not correct | 0 |
|  |  | Correct | 2 |
| 2 | Anatomical access point | Not correct | 0 |
|  |  | correct | 3 |
| 3 | Topical desinfection | Not used | 0 |
|  |  | Used | 1 |
| 4 | Gloves | Absence of gloves | 0 |
|  |  | Sterile gloves | 1 |
| 5 | Local anaesthetic | Not used | 0 |
|  |  | Used (awake patient) | 1 |
| 6 | Insertion of cannula | Insertion while drilling | 0 |
|  |  | Insertion until bone contact without drilling | 1 |
| 7 | Choice of cannula size | Canuala exceeds skinsurface more than 1 cm | 0 |
|  |  | Cannula exceeds surface of skin less than 1 cm | 1 |
| 8 | Angle of insertion (prove after completion) | Oblique angle | 0 |
|  |  | 90 degrees to surface of the skin | 2 |
| 9 | Depth of insertion | Cannula exceeds skin surface more than 1 cm | 0 |
|  |  | Cannula exceeds surface of skin less than 1 cm | 2 |
| 10 | Handing / drilling | Not correct drilled | 0 |
|  |  | 1 cm advancement of the cannula drilling after visible loss of resistance | 1 |
| 11 | Aspiration of bone marrow | Not performed | 0 |
|  |  | performed | 1 |
| 12 | Injection of local anaesthetic | Not performed | 0 |
|  |  | Performed | 1 |
| 13 | Flushing with NaCl 0.9% | Not performed | 0 |
|  |  | Performed | 2 |
| 14 | Fixation of cannula | Not considered | 0 |
|  |  | Considered | 1 |
| 15 | Marking of patient with a batch | Not considered | 0 |
|  |  | Considered | 1 |
| Maximum sum: 20 | | | |

**Fokus group round 4**

The new score was reviewed and the technique of the videorecording was tested to be applicable in the same two volunteers. All items of the adapted score were proven to be evaluable using the camera perspective described above.

**Fokus group round 5**

The authors observed the videorecords of two more volunteers and reviewed the weighting of the items. Two were adapted, then the final score was defined (Appendix B).
